# Identification of pyroptosis-related genes and long non-coding RNAs signatures in osteosarcoma

**DOI:** 10.1186/s12935-022-02729-1

**Published:** 2022-10-16

**Authors:** Jian Zhang, Jianjian Deng, Rui Ding, Jinghong Yuan, Jiahao Liu, Xiaokun Zhao, Tianlong Wu, Jingyu Jia, Xigao Cheng

**Affiliations:** 1 Department of Orthopedics, The Second Affiliated Hospital of Nanchang University, Nanchang, 330006 Jiangxi China; 2 Institute of Orthopedics of Jiangxi Province, Nanchang, 330006 Jiangxi China; 3 Institute of Minimally Invasive Orthopedics, Nanchang University, Jiangxi, 330006 China

**Keywords:** Osteosarcoma, Pyroptosis, lncRNA, Biomarkers, Prognostic signature

## Abstract

Osteosarcoma is a highly malignant tumor, with very high disability and fatality rates. However, the overall prognosis is not optimistic. Pyroptosis is a newly discovered cell death modality accompanied by inflammation, which is closely related to varieties of cancers. In this study, the RNA-seq data were downloaded from public databases, the differences in the expression of the pyroptosis-related genes (PRGs) were identified, and the six PRGs signature was established through the univariate and LASSO Cox analysis. The patients were grouped according to the PRGs signature, and the prognosis between the two groups was further compared. In addition, a ten pyroptosis-related lncRNAs (PRLs) prognostic signature was also constructed. Through functional analysis of the differentially expressed genes (DEGs), the immune-related pathways were found to be enriched. The Pearson correlation analysis showed a strong correlation between the pyroptosis-related biomarkers. Finally, we identified a promising biomarker, CHMP4C, which is highly expressed in osteosarcoma. Overexpression of CHMP4C promoted the proliferation, migration and invasion of the osteosarcoma cell. Our results thus provide new evidence for exploring prognostic biomarkers and therapeutic targets of osteosarcoma.

**Supplementary Information:**

The online version contains supplementary material available at 10.1186/s12935-022-02729-1.

## Introduction

Osteosarcoma is an osteogenic malignant tumor originating in the bone tissue and is most frequent in adolescents[1, 2]. It has a high degree of malignancy, low sensitivity to radiotherapy and chemotherapy, easy recurrence and metastasis, and a poor prognosis[3]. The current main treatment includes a combination of neoadjuvant chemotherapy and extensive surgical resection, but it still has a low overall survival rate[4]. Therefore, investigating novel early diagnosis and prognostic indicators is of great significance for patients with osteosarcoma.

Pyroptosis is a type of cell programmed inflammatory death different from apoptosis[5]. It relies on the activation of some caspases and is accompanied by the lysis of GSDMD and the release of pro-inflammatory cytokines[5, 6]. Finally, stimulating the innate immune mechanism, expands the inflammatory response, causing the cells to collapse and die[7]. Pyroptosis is activated by the Caspase-1-mediated classical pyroptosis pathway activated by the inflammasomes and the non-Caspase-1-mediated pyroptosis pathway[8–10]. Pyroptosis can form an inflammatory microenvironment through the pro-inflammatory effects, or affect certain signaling pathways to promote the growth of the malignant tumors[11, 12]. However, many studies have confirmed that pyroptosis playing a key role in malignant tumor treatments by regulating the activity of a certain target or signal pathway[13, 14]. For example, Hou et al. have found that PD-L1 can regulate the expression of gasdermin C, transforming apoptosis into pyroptosis, and promote tumor necrosis[15]. However, the specific function of pyroptosis in the prognosis and treatment of osteosarcoma is still at its infancy.

In this study, a systematic study of the PRGs was conducted in osteosarcoma and 6 PRGs signature were identified to have powerful prognostic functions and verified in the GSE21257 cohort. The relationship between the PRGs risk model and the immune microenvironment has also been discussed. In addition, a 9 PRLs signature was also found to be related to the prognosis of osteosarcoma. Through functional enrichment analysis, the possible mechanism of action was discussed. Compared with osteoblasts, the expression level of CHMP4C in osteosarcoma cells was up-regulated, which might be a promising biomarker. Finally, overexpression of CHMP4C promoted the proliferation, migration and invasion of the osteosarcoma cell line U2OS. Our findings provide new evidence for exploring the prognostic biomarkers and therapeutic targets of osteosarcoma.

## Materials and methods

### Data collection

The RNA-seq data and clinical information of 85 osteosarcoma patients were screened from the TCGA database (TARGET-OS project). The gene expression data of the musculoskeletal samples from 396 healthy humans were collected from the GTEx (The Genotype-Tissue Expression) database. To eliminate the platform data difference between the TCGA and GTEx databases, the gene transcriptional expression data of each sample were transformed into log2 (FPKM value + 1).

The GSE21257 and GSE42352 dataset of osteosarcoma was obtained from the high-throughput microarray expression profile database (Gene Expression Omnibus database, GEO, https://www.ncbi.nlm.nih.gov/geo/)). GSE21257 contained the gene expression data and related clinical information of 53 osteosarcoma patients, which was used as the verification cohort. GSE42352 contained 15 normal samples and 103 osteosarcoma samples for analyzing the differential expression.

A total of 57 PRGs were collected from previous articles [14, 16–18] and MSigDB (http://www.gsea-msigdb.org/gsea/msigdb/), as shown in Additional file 2: Table S3. Analyzed the interaction between the PRGs by the STRING online tool (http://www.string-db.org/).

### Differential analysis

Using the “limma” package, FDR < 0.05 and logFC > 1 as screening criteria, the differences of PRGs expression between the osteosarcoma and normal samples in the combination of TARGET and GTEx cohorts were determined, and the differences in the PRGs expression were visualized. The expression levels of CHMP4C were visualized in several common cancers by the GEPIA online tool (https://cistrome.shinyapps.io/timer/) and TIMER online tool (Gene Expression Profiling Interactive Analysis, http://gepia.cancer-pku.cn /).

### Construction and validation of the PRG-based prognostic signature

The univariate Cox regression analysis of 57 PRGs was carried out by using the “survival” R package in the TARGET cohort, where *p* < 0. 05 is considered to be related to prognosis. The genes obtained from the univariate Cox regression analysis were analyzed by “glmnet” R package for 1000 times iterative Lasso regression analysis, and the final key prognostic genes were determined.

To obtain the PRLs, the 57 PRGs were compared with the lncRNAs one by one to calculate the Pearson correlation coefficient in the TARGET database. The PRLs were screened according to the absolute value of correlation coefficient ≥ 0.4 and *p* < 0.05. Then, to build a PRLs prognostic model, the differentially expressed PRLs were selected, the prognostic PRLs were screened by univariate Cox regression, and the PRLs prognostic signature was constructed including ten PRLs by LASSO Cox analysis, at the same time, the risk coefficient of each gene was obtained. A risk scoring equation based on the expression of the genes was constructed:

$$Risk \,score={\sum }_{i = 1}^{n}\left({Coef}_{i}*{x}_{i}\right)$$

Here, ${Coef}_{i}$refers to the regression coefficient of the gene, and ${x}_{i}$ is the expression level of the gene.

### Evaluation and verification of the risk model

The risk score of the osteosarcoma samples was calculated, ranked from low to high, and the osteosarcoma samples were divided into the low-risk and high-risk groups according to the median. The Kaplan—Meier curve was used to analyze the difference in the prognosis between the groups. The time-dependent ROC curve was drawn by the “survival” R package. The univariate Cox and multivariate Cox regression analyses were used to explore the independent prognostic factors, including age, gender, and metastasis. The “rms” package was used to establish a nomogram, and draw calibration curves to assess the consistency of the predicted results with the actual results.

### Immune cell infiltration and immune score analysis

The ssGSEA was used to evaluate the immune cell infiltration in each sample. Based on the ESTIMATE algorithm, the ESTIMATE score, immune score, and stromal score of the osteosarcoma patients were calculated by the “estimate” R package.

### Functional enrichment analysis

The DEGs between the low-risk and high-risk groups were determined using the “limma” package, and the “clusterProfiler” R package was used for Gene Ontology (GO) and KEGG analysis. The hallmark gene sets (h.all.v7.4.symbols) of the high- and low-risk groups were further analyzed by the GSEA software, and a gene enrichment map was drawn. The GSEA software was downloaded from (http://www.gsea-msigdb.org/).

### Cell lines and reagents

The hFOB1.19 and the 143B, SaOS2, and U2OS osteosarcoma cell lines were purchased from the National Collection of Authenticated Cell Cultures (Shanghai, China). The TRIzol reagent and penicillin/streptomycin were purchased from Thermo Fisher Scientific, USA. The RT-qPCR kit was purchased from Takara Company, Japan. The Dulbecco’s modified Eagle’s medium (DMEM) and fetal bovine serum (FBS) were purchased from Gibco, USA. The primers (CHMP4C, GAPDH) were purchased from Sangon Biotech Shanghai, China. The Primers are listed in Additional file 1: Table S4.

### Cell culture

The osteosarcoma cell lines were grown in complete DMEM (containing 10% FBS and 1% penicillin/streptomycin) at 37℃ in a humidified atmosphere containing 5% CO_2_. The osteoblast cell lines were grown in the same complete medium at 34℃ in a humidified atmosphere containing 5% CO_2_.

### Clinical specimens

We collected 3 osteosarcoma tissues and 3 matched adjacent normal tissues. The samples came from patients who underwent surgery at The Second Affiliated Hospital of Nanchang University and were pathologically diagnosed with osteosarcoma. All patients signed an informed consent form, and the study was approved by the Research Ethics Committee of the Second Affiliated Hospital of Nanchang University.

### RNA extraction and RT-qPCR

Add TRIzol to the cells to extract total RNA, and obtain cDNA after reverse transcription. The qPCR kit was used to detect the expression of CHMP4C using the relative quantification method according to the instructions and GAPDH as an internal control.

### Lentivirus infection

Lentiviruses containing pFBLV-CHMP4C-Puro and controls were purchased from Focus Bioscience Company (Nanchang, China), and U2OS cells were infected according to the manufacturer’s protocol. Puromycin (1.0 µg/mL) was used to select stably transfected cells. Overexpression of CHMP4C was confirmed via western blotting.

### Western blotting

Proteins were extracted from cells using RIPA lysis buffer and quantified using the BCA method. The proteins were separated by 10% SDS-PAGE gel electrophoresis and transferred to PVDF membrane. Block with 5% skim milk and incubate with anti-CHMP4C (Abcam) overnight at 4℃. The next day, the membrane was rinsed twice with PBST and incubated with horseradish peroxidase secondary antibody (1:20000) for 1 h. Finally, expression of the corresponding protein was observed via chemiluminescence and analyzed using ImageJ software.

### CCK-8 and colony formation assays

The proliferation of osteosarcoma cells was detected by CCK-8 and colony formation assays. For the CCK-8 assay, CHMP4C overexpressing cells and control U2OS cells were seeded in 96-well plates at a density of 1 × 103 cells/well in 5 replicates. CCK-8 reagent was added to the wells at the indicated time points. Plates were incubated at 37 °C for 1.5 h before recording optical density (OD) at 450 nm.

In colony formation experiments, U2OS cells were seeded into 6-well plates at a density of 1 × 103 cells/well. Cells were cultured for 2 weeks, and the medium was changed every 3 days. After 2 weeks, Colonies were fixed and stained with 1% crystal violet. The plates were photographed and the number of cell colonies in each well was counted.

### Wound healing and transwell invasion assays

The migration and invasion abilities of osteosarcoma cells were evaluated by wound healing and transwell migration and invasion assays. In wound healing assays, transfected osteosarcoma cells were seeded into six-well plates. When the cell density reached about 90%, the cells were scratched with a 10 µL sterile pipette tip to allow intercellular space to form, and cultured in serum-free medium for 48 h. An inverted microscope was used to observe the gaps at 0, 24 h, and 48 h and take pictures.

Transwell invasion assays were performed using Falcon® Cell Culture Inserts (NY, USA). Transfected osteosarcoma cells were digested and resuspended in serum-free medium at a density of 105 cells/mL. 400 µL of cell suspension was added to the upper chamber and 700 µL of medium (10% fetal bovine serum) was added to the lower chamber. After 24 h, the cells in the bottom cavity were fixed, stained with 1% crystal violet, and photographed with an inverted microscope.

### Immunohistochemical staining

To further verify the expression of CHMP4C, immunohistochemistry was performed on paraffin sections following the standard protocol (Abcam, ab272638). All slides were observed and photographed under XSP-C204 microscope (CIC).

### Statistical analysis

Analyzed the data with the R Software (v4.0.4) and GraphPad Prism (v9.0). The student’s t-test was used to compare the differences between the two groups. *p* < 0.05 indicated that the difference was statistically significant.

## Results

### Defining the PRGs expression patterns in osteosarcoma

**Fig. 1:**
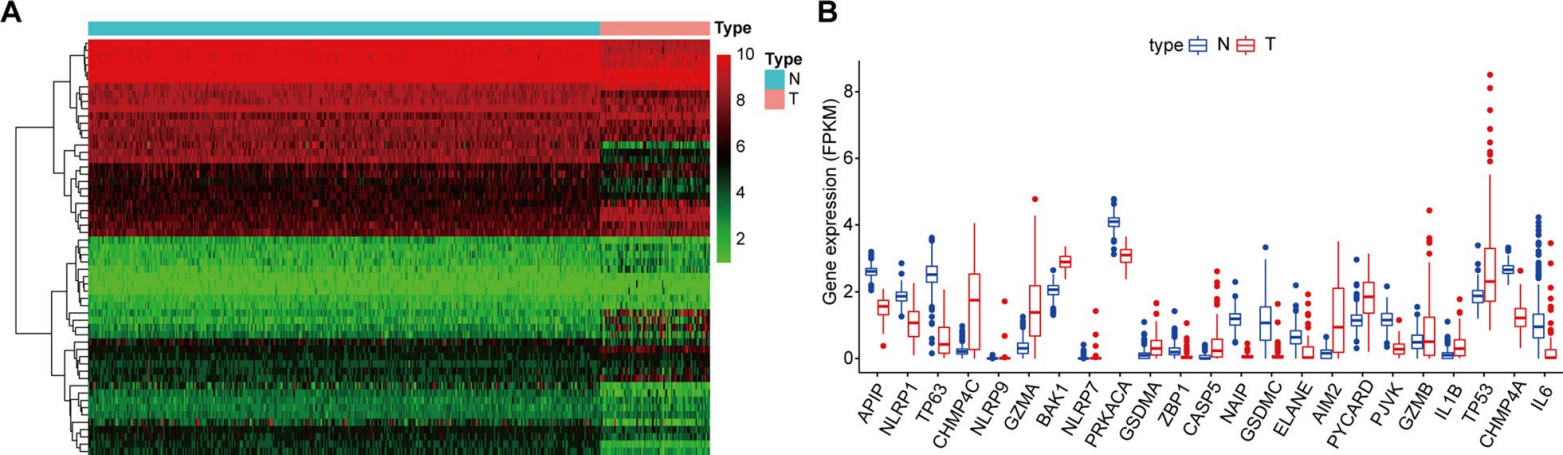
Expression of the pyroptosis-related genes (PRGs) in osteosarcoma.** A** The heatmap showed the expression levels of 57 PRGs in normal and tumor samples. **B** The boxplot of 23 differentially expressed PRGs (logFC > 1) between the normal and the tumor tissues

The expression of 58 PRGs was first explored in the osteosarcoma and normal musculoskeletal tissues using a combination of TARGET and GTEx datasets. The heat map showed the expression patterns of 58 PRGs (Fig. 1A). The boxplot (Fig. 1B) further demonstrated the expression levels of the differentially expressed PRGs (logFC > 1, FDR < 0.05). We also constructed a PPI network. Additional file 4: Fig. S2 shows the interaction between the PRGs.

### Establishment and evaluation of the PRGs prognostic signature

**Fig. 2:**
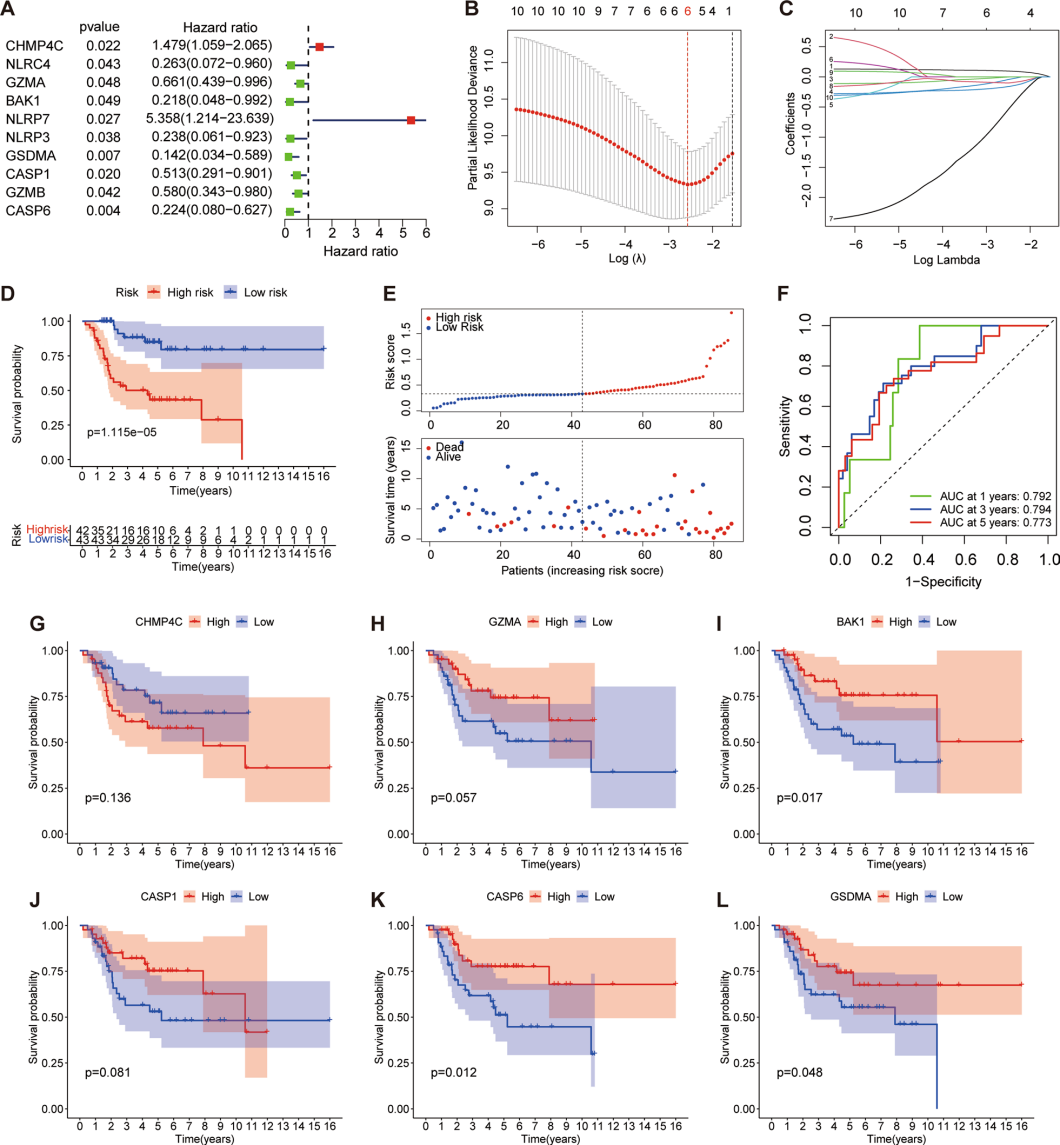
Construction of PRGs signature in the TARGET cohort.** A** Univariate Cox regression showed 10 PRGs related to osteosarcoma survival (P < 0.05). **B** LASSO analysis of 10 prognostic PRGs. **C** Cross-validation for tuning parameter selection in the LASSO regression. Coefficients refer to the risk coefficient corresponding to each gene. **D** Kaplan-Meier survival curve based on osteosarcoma patients from the TARGET cohort. **E** Distribution of risk score, survival status of the six PRGs signature. **F** The time-dependent ROC analysis of the signature. **G–L** Kaplan-Meier survival analysis of single gene in the TARGET cohort

In the TARGET data set, 58 PRGs were included in the univariate Cox regression analysis, and 10 PRGs were determined to be related to the prognosis of osteosarcoma patients (Fig. 2A). By LASSO Cox regression analysis, six key PRGs (Fig. 2B, C) were further identified, establishing the prognosis model of osteosarcoma. The specific information of each gene was shown in Additional file 1: Table S1. The survival curve showed that the PRGs signature can clearly distinguish between high- and low-risk groups of patients (Fig. 2D, p < 0.001). With the increase of the risk score, the death rate of the patients increased, as shown in the scatter plot (Fig. 2E). The area under the curve (AUC) of the 1-, 3-, 5-year overall survival rates were 0.792, 0.794, 0.773, respectively (Fig. 2 F). PCA analysis showed that significant differences in the distribution of patients (Additional file 3: Fig. S1A, B). Through the survival analysis of the single gene, the BAK1, CASP6, and GSDMA were found to be linked to the prognosis of osteosarcoma (Fig. 2G–L).

### Verification of the PRGs signature

**Fig. 3:**
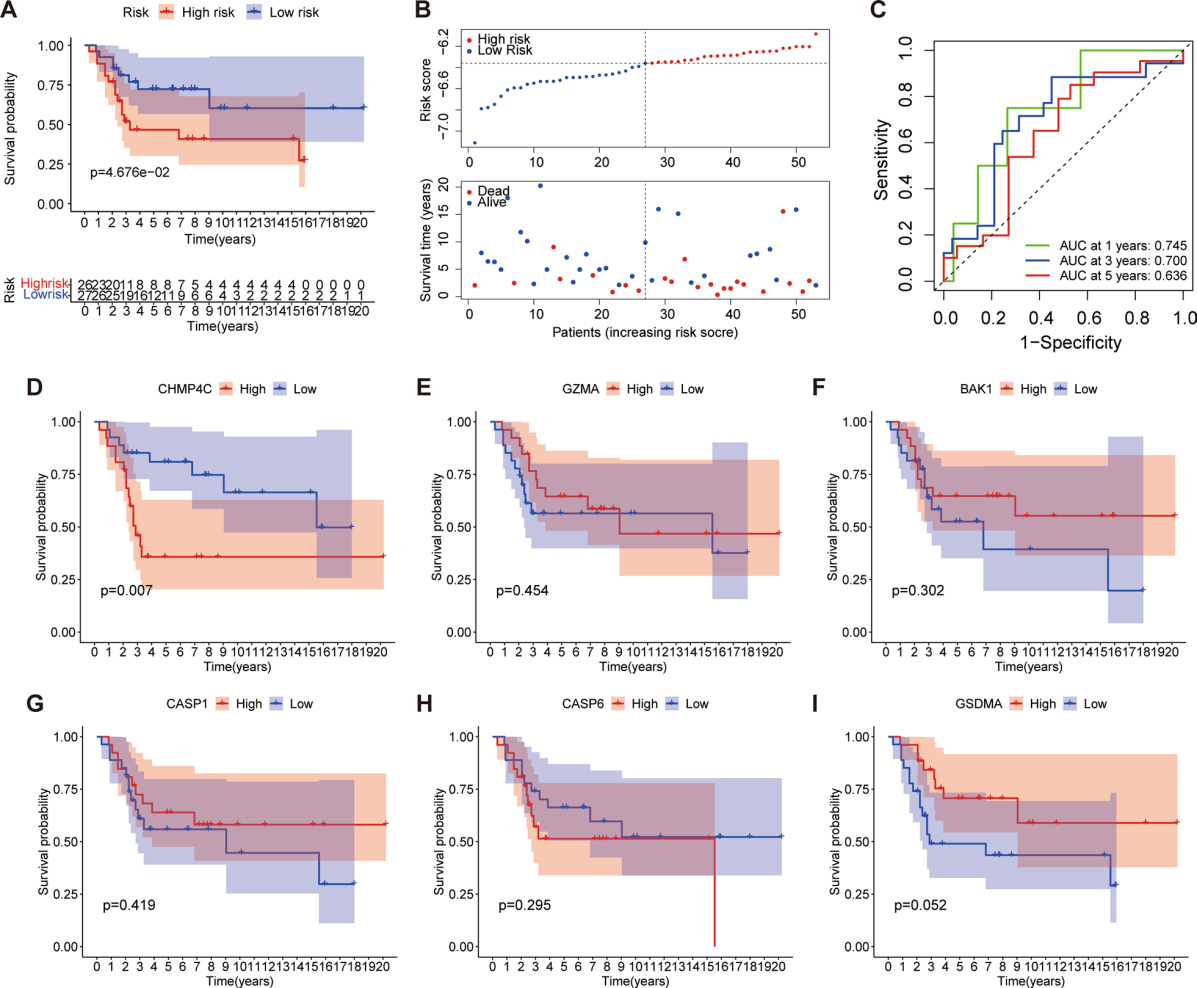
Validation of the PRGs signature in the testing set.** A** Kaplan-Meier survival curve analysis based on osteosarcoma patients from the GSE21257 cohort. **B** Distribution of risk score, survival status. **C** The time-dependent ROC analysis. **D–I** Kaplan-Meier survival analysis of single gene in the GSE21257 cohort

To verify the six-gene prognostic signature, we applied the six-gene model to the GSE21257 cohort, and the survival analysis of the verification group was performed (Fig. 3A, B), and the results are consistent with the training cohort. The 1-, 3-, and 5- years AUC was found to be 0.745, 0.700, 0.636, respectively (Fig. 3C). The survival analysis of a single modeling gene was also consistent with the trend of TARGET (Fig. 3D–I). The result indicated that the six PRGs signature has a good predictive effect on the external data set.

### The relevance of clinical features and PRGs prognostic signature

**Fig. 4:**
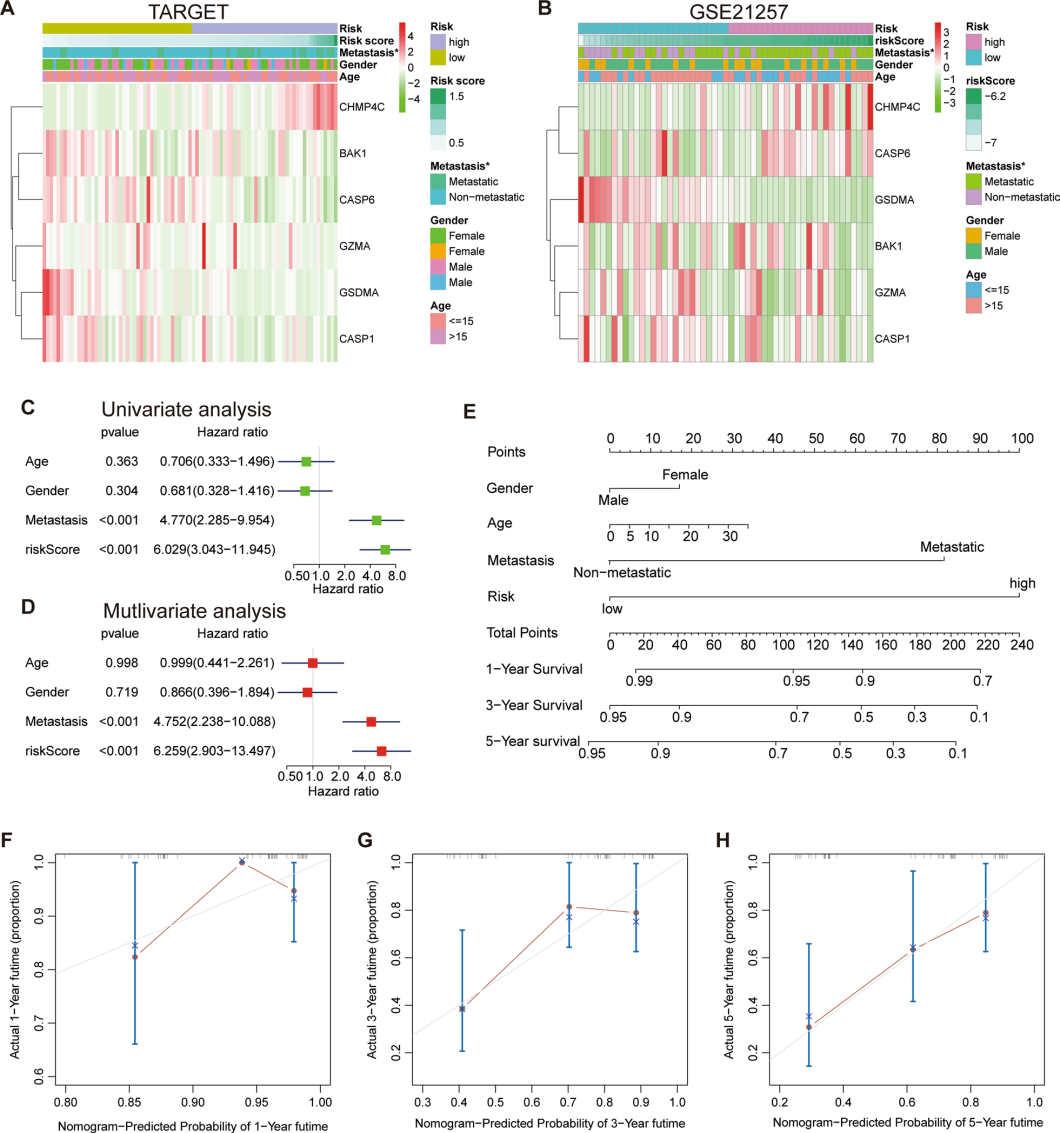
Combination of the six PRGs signature and clinical features.** A**,** B** Heatmap and the clinical characters of the two groups (*p < 0.05). **C**,** D** Univariate and multivariate Cox regression analysis for independent prognostic analysis of risk model. **E** Nomogram based on age, gender, metastasis, and risk in the TARGET database. **F–H** The nomogram calibration curves for predicting 1-, 3-, and 5-year survival in the TARGET cohort

The heat map was drawn to explore the relevance of the various clinical characteristics and the PRGs signature, including age, sex, and metastatic status (Fig. 4A, B). The expression level of CHMP4C was found to positively correlate with the risk score, while GSDMA was found to negatively correlate with the high risk of osteosarcoma, suggesting that CHMP4C may be a risk factor. At the same time, the high risk was found to have a high correlation with osteosarcoma metastasis, and there were statistical differences in the training and validation sets. The box plot was drawn to visualize the correlativity between the metastasis and the risk score (Additional file 3: Fig. S1C, D). However, there were no gender and age differences between the two subgroups. The univariate and multivariate Cox regression analysis showed that the risk score can be used to affect the prognosis of the osteosarcoma patients when other clinical factors were considered (Fig. 4C, D). To evaluate the prognostic ability of PRGs, we selected the clinical variables, including gender, age, metastasis, and risk score as the parameters for establishing a nomogram based on the training cohort (Fig. 4E). The nomogram model was evaluated using a C index of 0.809 and a 95% confidence interval of 0.725 to 0.893. The calibration curves results indicated that the nomogram was superior in predicting the prognosis of the osteosarcoma patients.

### Immune cell infiltration and immune score

**Fig. 5:**
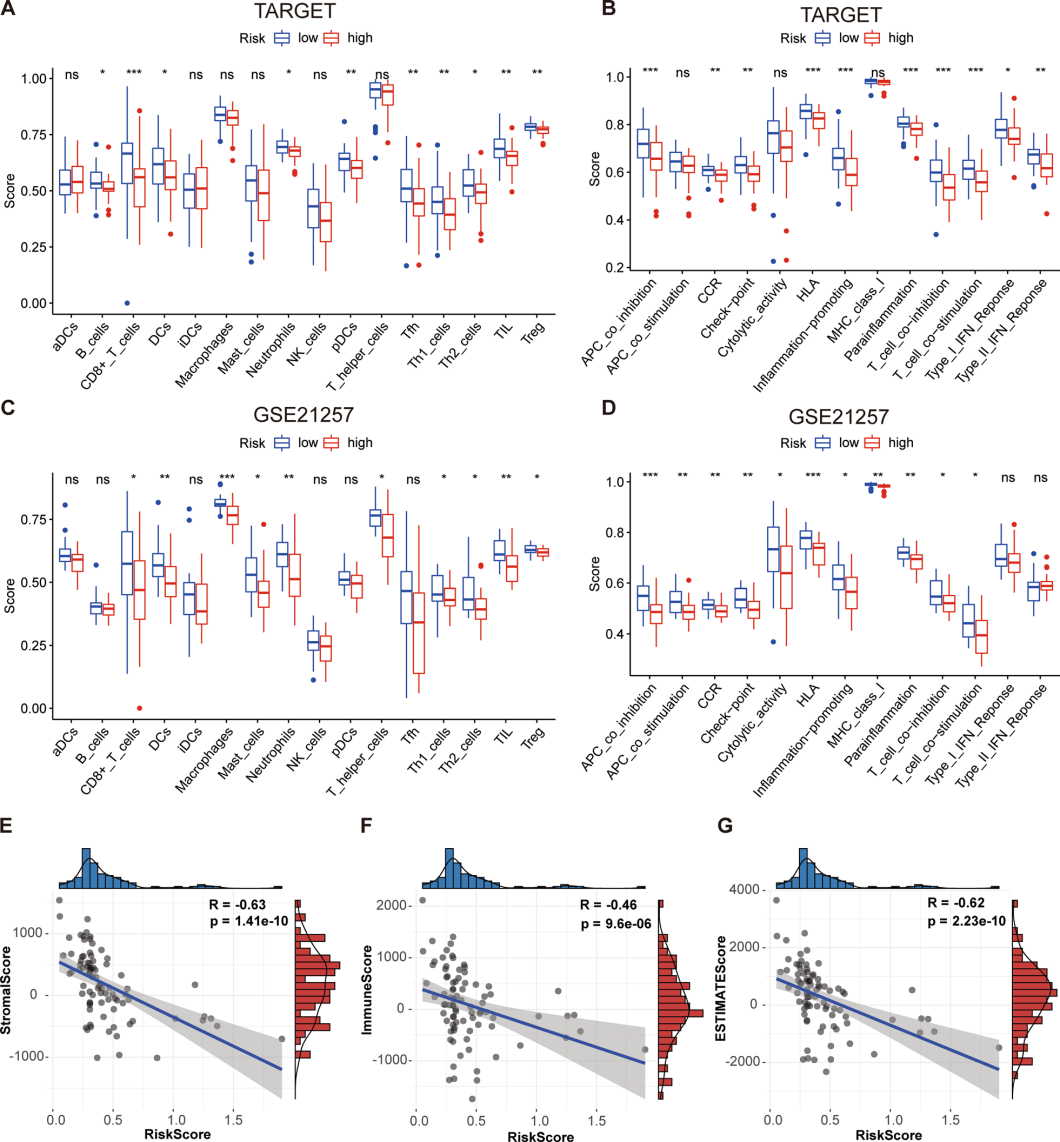
Distribution and visualization of immune status.** A**,** B** Relationship between risk score and immune cell infiltration and related functions via ssGSEA analysis. **E–G** Pearson correlation analysis shows that the risk score is significantly related to the Stromal score, immune score, and ESTIMATE Score calculated by the ESTIMATE algorithm. *P < 0.05, **P < 0.01 and ***P < 0.001

Based on the TARGET data set, the ssGSEA (single sample gene set enrichment analysis)[19] was performed to evaluate the values of immune cell infiltration. As the box plot shown in Fig. 5A–D, the immune cell infiltration and related functions showed a downward trend in the high-risk group. To further explore the correlation of the immune status and risk score, we used ESTIMATE to calculate the stromal cell score, immune cell score, and ESTIMATE score of each sample. The risk scores showed a significant negative correlation with the stromal score (Fig. 5E), immune score (Fig. 5F), and ESTIMATE score (Fig. 5G). This indicated that the high-risk samples were found to contain a smaller number of immune and stromal cells.

### Identification of the PRLs and establishment of the signature

**Fig. 6:**
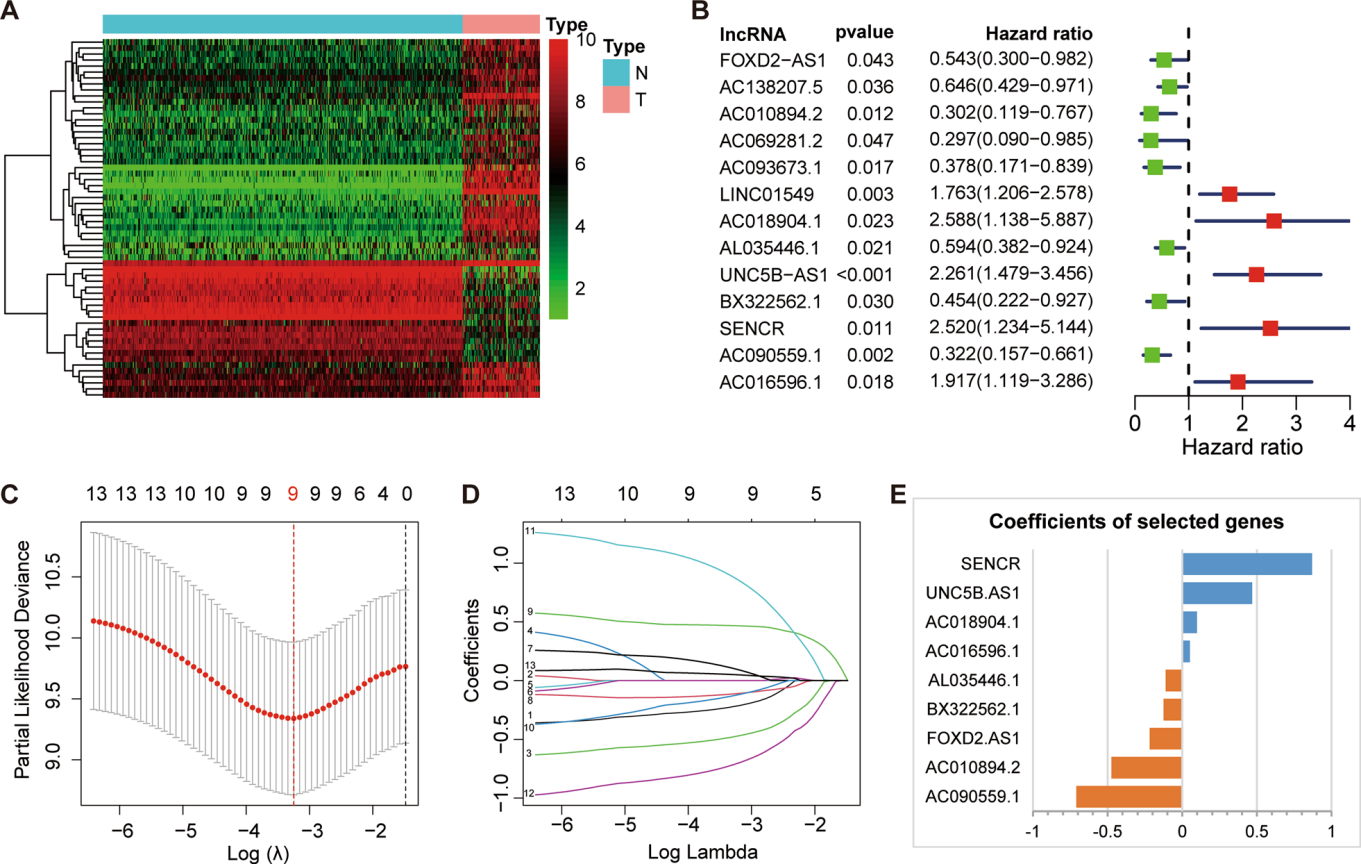
Construction of pyroptosis-related lncRNAs (PRLs) signature.** A** Heatmap of the PRLs between the normal and the tumor tissues. **B** Univariate Cox regression showed 13 PRLs related to osteosarcoma survival (P < 0.05). **C, D** Lasso regression for PRLs in univariate Cox regression. Coefficients refer to the risk coefficient corresponding to each gene. **E** The coefficients of the nine PRLs.

First, we analyzed the lncRNA data from the TARGET and GTEx databases and identified 13,012 lncRNAs. Then, the Pearson correlation analysis was used in the TARGET database to screen out 302 PRLs. By the “limma” package, we obtained 60 PRLs were differentially expressed between the osteosarcoma samples and normal samples, including 44 up-regulated lncRNAs and 16 down-regulated lncRNAs, results are shown in a heat map (Fig. 6 A). Combining these differentially expressed PRLs with the corresponding clinical information from TARGET, 13 lncRNAs related to the prognosis of osteosarcoma were initially screened (Fig. 6B), and 9 key lncRNAs (FOXD2-AS1, AC010894.2, AC018904.1, AL035446.1, UNC5B-AS1, BX322562.1, SENCR, AC090559.1, AC016596.1) were further determined through the LASSO regression analysis (Fig. 6C, D, E, Additional file 1: Table S2).

### Validation of the PRLs prognostic signature

**Fig. 7:**
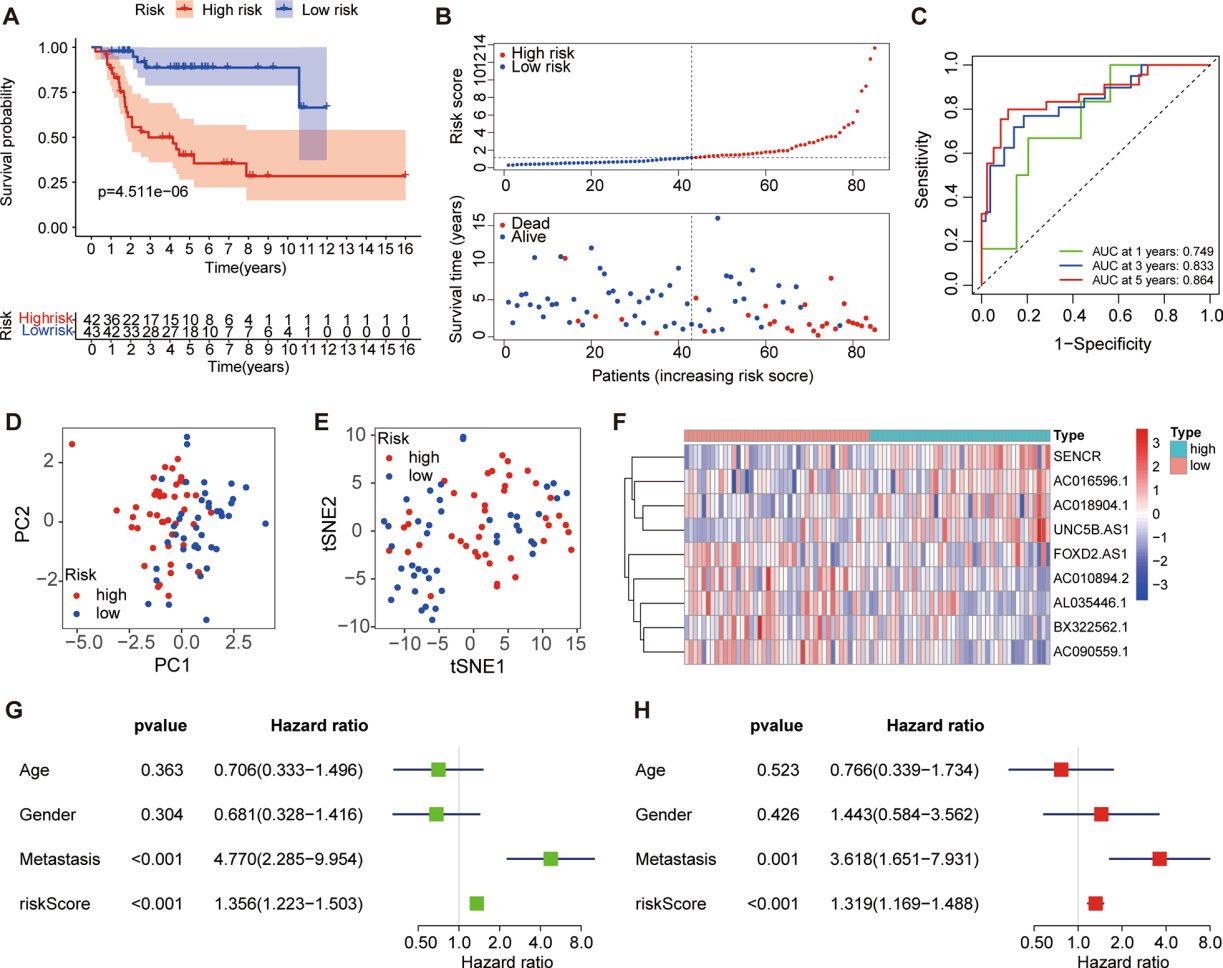
Prognostic analysis of the PRLs signature.** A** Kaplan–Meier survival curve analysis of patients in the high-risk group and low-risk group. **B** The distributions of survival status and risk score. **C** The AUC of time-dependent ROC curves. **D**,** E** PCA plot and t-SNE analysis based on the PRLs signature. **F** Heatmap of the six PRLs between the high and low-risk group. **G**,** H** Univariate and multivariate Cox analysis for the risk score

The Kaplan–Meier curve showed that PRLs prognostic model can distinguish patients in different groups (*p* < 0.001) (Fig. 7A, B). The time-dependent ROC curve was used to evaluate the performance of the gene signature to predict overall survival. The AUC values for 1, 3, and 5 years are 0.732, 0.701, and 0.695, respectively (Fig. 7C). The PCA and t-SNE analysis showed significant differences in the distribution of patients (Fig. 7D, E). According to the risk heat map, SENCR, AC016596.1, AC018904.1, and UNC5B.AS1 was suggested to be high-risk PRLs.

### Functional analysis and gene set enrichment analysis (GSEA)

**Fig. 8:**
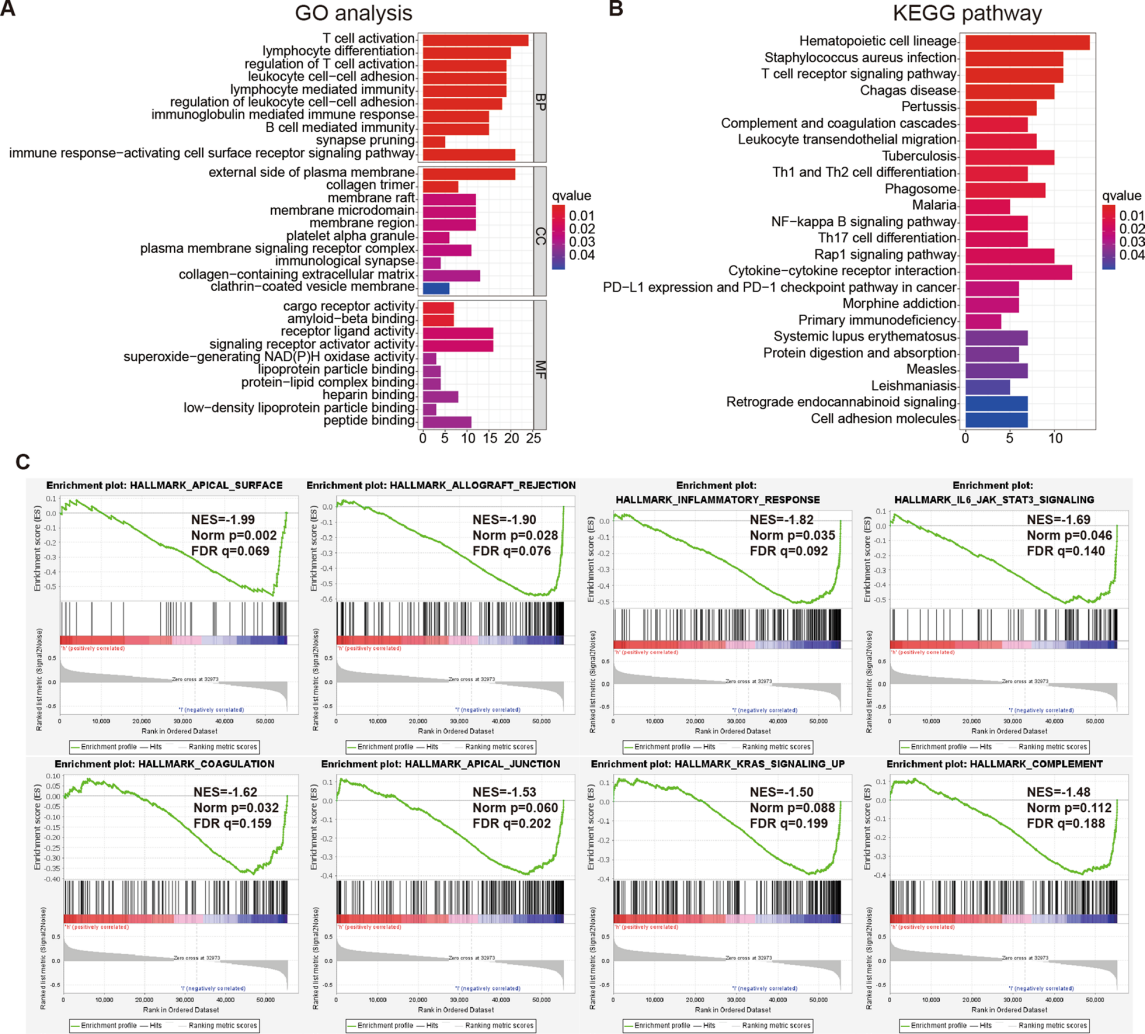
Functional enrichment analysis between low- and high-risk osteosarcoma patients based on the PRLs signature.** A** Gene ontology analysis of DEGs between low- and high-risk osteosarcoma patients. **B** KEGG pathway analysis of DEGs between low- and high-risk osteosarcoma patients. **C** Gene set enrichment analysis (GSEA) of hallmarks enriched in low-risk osteosarcoma patients

To study the differences in the molecular biological mechanisms between the groups, functional analysis was used to analyze the DEGs in the TARGET cohort. In biological processes, DEGs are mainly involved in T cell activation and lymphocyte differentiation (Fig. 8A). Among the cellular components, the term enrichment is mainly related to the external side of the plasma membrane and collagen trimer (Fig. 8A). In terms of molecular functions, the rich terms are mainly related to the cargo receptor activity and signaling receptor activator activity (Fig. 8A). The Kyoto Encyclopedia of Genes and Genomes (KEGG) analysis revealed that DEGs are mainly enriched in the T cell receptor signaling pathway pathways (Fig. 8B).

The tumor characteristics and related pathways were studied by the GSEA software. Several tumor-related markers, including the KRAS signaling pathway, IL-6/JAK/STAT3 signaling pathway, and inflammatory response were identified, which were enriched in low-risk patients (Fig. 8C). The immune-related biological processes like a complement, coagulation, and apical surface were also enriched in the low-risk patients (Fig. 8C).

### Relationship between the expression of the pyroptosis-related prognostic markers

**Fig. 9:**
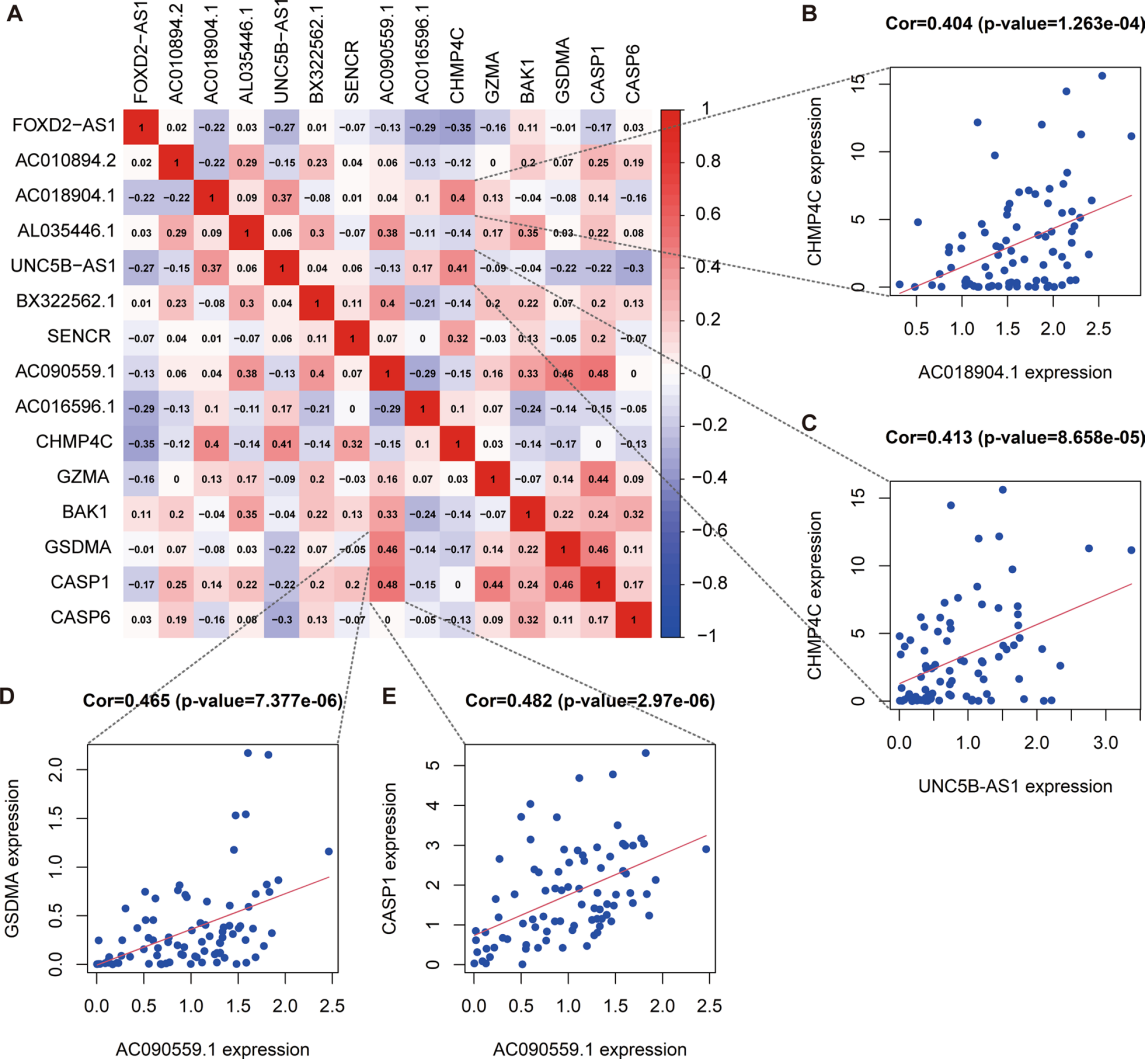
The correlation analysis of prognostic pyroptosis-related biomarkers.** A** Heatmap of Pearson correlation between the pyroptosis-related biomarkers. The x-axis and y-axis represent genes. Red blocks represent positive correlation, and blue blocks represent negative correlation. **B–E** Representative results of correlation analysis. Cor: correlation coefficient

To better understand the correlation between PRLs and PRGs, a Pearson correlation analysis was conducted. In the correlation analysis, the expression of the lncRNA AC090559.1 and CASP1, GSDMA were positively related, and the CHMP4C expression level showed a positive correlation with that of AC018904.1 and UNC5B-AS1 (*r* ≥ 0.4, *p* < 0.05). The result is shown in Fig. 9.

## Validation of the expression level of CHMP4C

**Fig. 10:**
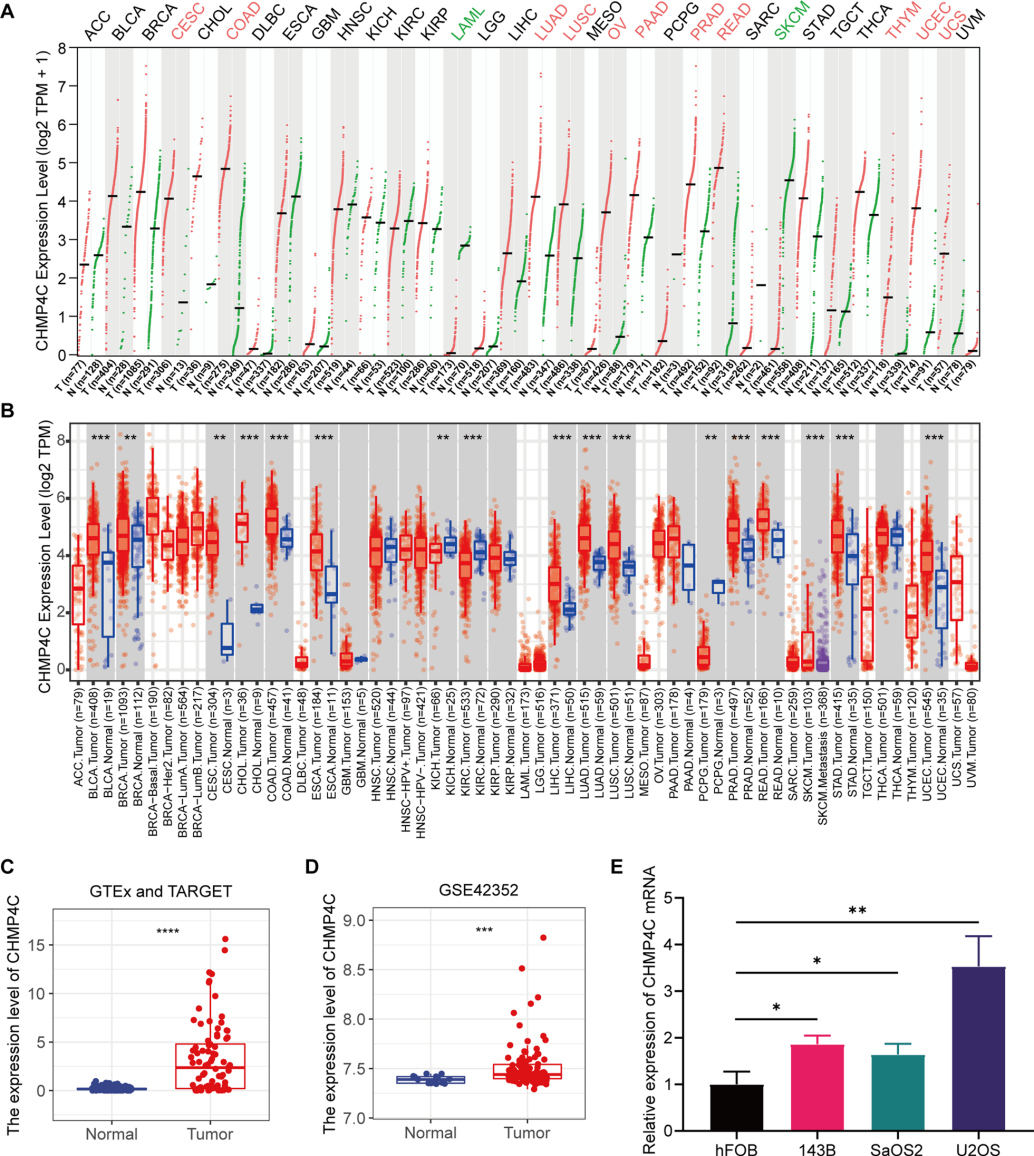
The expression levels of CHMP4C.** A** Pan-cancer analysis of CHMP4C expression based on the GEPIA database. **B** Pan-cancer analysis of CHMP4C expression based on TIMER database. **C** The CHMP4C expression level (FPKM) in osteosarcoma and normal tissues, based on the combination of GTEx and TARGET. **D** The CHMP4C expression level in osteosarcoma and normal tissues, based on the GSE42352 cohort. **E** The qRT-PCR result of CHMP4C in hFOB, 143B, SAOS2, U2OS cell lines. *P < 0.05, **P < 0.01 and ***P < 0.001

CHMP4C belongs to the family of charged multivesicular body protein (CHMP). Recent studies have demonstrated a human polymorphism in CHMP4C to be associated with the increased risk for several other cancers [20], and CHMP4C can also regulate the proliferation of the tumor cells through the cell cycle pathway [21]. The Pan-Cancer analysis showed that CHMP4C to be up-regulated in breast cancer, colon adenocarcinoma, liver hepatocellular carcinoma, lung adenocarcinoma, and other malignant tumors (Fig. 10A, B), and the Kaplan–Meier analysis shows that CHMP4C may be a risk factor for lung adenocarcinoma, pancreatic ductal adenocarcinoma and thymoma, moreover, univariate Cox analysis showed that CHMP4C could be used as an independent prognostic factor (Additional file 3: Fig. S1H). However, CHMP4C has not been described in osteosarcoma. The analyses of data from the GTEx and TARGET collections and GSE42352 in Fig. 10C and D showed the expression of CHMP4C, to be significantly up-regulated in the osteosarcoma samples. In addition, the expression of CHMP4C was quantified in the osteoblasts and osteosarcoma cell lines. The RT-qPCR showed that CHMP4C mRNA expression levels in the osteosarcoma cells were significantly increased compared to the osteoblasts (Fig. 10E). Then, we used immunohistochemical staining to explore the differential expressions of CHMP4C in tumor and adjacent normal tissues (Additional file 3: Fig. S1I).

### CHMP4C suppressed Osteosarcoma Cell Proliferation Migration, and Invasion

**Fig. 11:**
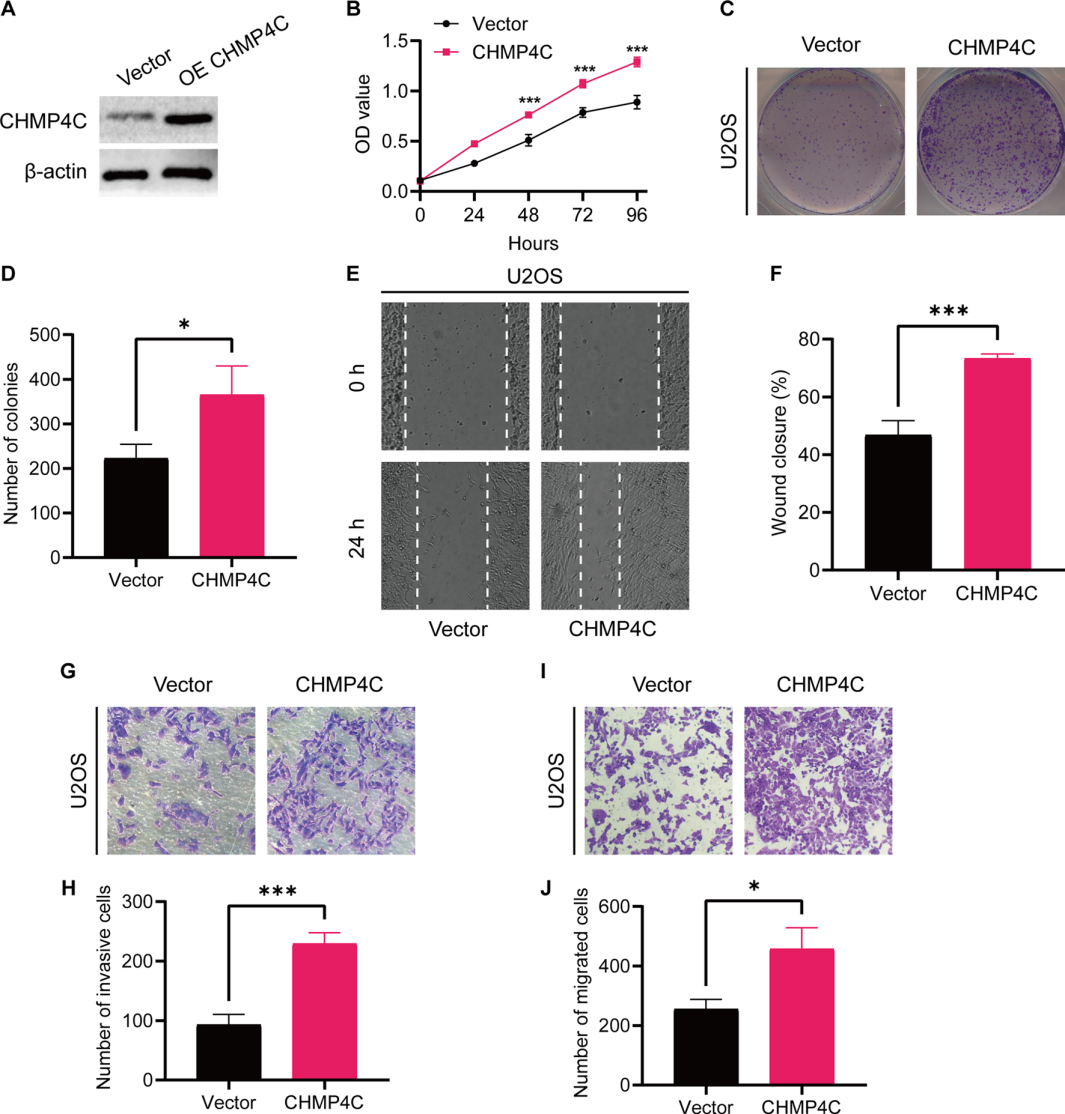
The effect of CHMP4C on osteosarcoma cell proliferation, migration, and invasion.** A** Protein expression levels of CHMP4C were measured by western blot analysis. **B–D** CCK-8 and colony formation assays were used to assess the osteosarcoma cell proliferation. **E, F** The wound healing assay was performed to estimate the effect of CHMP4C overexpression on cell migration. Scale bar, 0.2 mm. **G–J** The transwell assay was conducted to assess the effect of CHMP4C overexpression on osteosarcoma cell invasion and migration. *P < 0.05, **P < 0.01 and ***P < 0.001

Since CHMP4C was expressed at the highest fold in U2OS, we selected the U2OS cell line for further experiments. We used lentiviral transfection to upregulate the expression of CHMP4C in U2OS cells (Fig. 11A), and then examined its effect on cell proliferation. As shown in Fig. 11B–D, CCK-8 and colony formation experiments showed that overexpression of CHMP4C resulted in down-regulated proliferation of U2OS cells. Wound healing experiments demonstrated that overexpression of CHMP4C significantly promoted the migration ability of U2OS cells (Fig. 11E, F). Furthermore, transwell experiments showed that CHMP4C overexpression significantly promoted the migration and invasion of U2OS cells (Fig. 11G–J). These results suggest that upregulation of the CHMP4C gene promotes the proliferation, migration and invasion of U2OS cells.

## Discussion

This study analyzed the differential expression of the PRGs between the osteosarcoma and healthy tissues. Then, 10 prognostic PRGs were preliminarily screened out through the univariate Cox regression analysis. By the Lasso Cox regression analysis, 6 key PRGs were screened for constructing the optimal model, namely CHMP4C, GZMA, BAK1, CASP1, CASP6, and GSDMA, and a six PRGs signature was successfully constructed for osteosarcoma. Compared with low-risk patients, the survival rate of high-risk patients is significantly lower. The results of the validation cohort also showed that the model has good prognostic significance. In addition, the risk scores and other clinicopathological factors (including age, gender, and metastasis) were used to construct an excellent nomogram for predicting the survival rates. In summary, these results confirmed that in our study, the six PRGs signature have a strong prognostic value in the patients with osteosarcoma and can be extended to other cohorts.

Pyroptosis is a new mechanism of programmed cell death, also known as gasdermin-mediated programmed necrotic cell death [5, 22, 23]. Recent studies have shown cell pyroptosis to be closely related to the occurrence and development of cancer [11, 24]. However, the role of pyroptosis in osteosarcoma remains unclear. Although some studies have recently reported pyroptosis-related signatures [25, 26], there are still some shortcomings in experimental verification, which affects the widespread application of signatures. This study identified 6 key PRGs related to the prognosis of osteosarcoma, and their role in tumors has been studied. The GZMA (Granzyme A) belongs to serine proteases, which are abundant in the cytotoxic T and NK cells [27, 28]. When GZMA is delivered to the target cells through the immunological synapse, it can activate pyroptosis [29, 30]. This immune effect mechanism promotes the cytotoxic T cell-mediated tumor clearance in the mice [29]. BAK1 (BCL2 Antagonist/Killer 1) belongs to the BCL2 family, which is located in the mitochondria and induces apoptosis [31, 32]. Recent studies have reported BAK1 to be involved in the caspase-3-GSDME mediated pyroptosis pathway, the knockdown of BAK1 can reduce cell pyroptosis [33]. CASP1 (caspase-1) and CASP6 (caspase-6) are both members of the cysteine-aspartic acid protease (caspase) family. The activation of caspase plays a central role in programmed cell death. The low expression of CASP1 is related to the poor prognosis of lung adenocarcinoma, and CASP1 inhibits the invasion and migration of the non-small cell lung cancer (NSCLC) cells [34]. Emerging pieces of evidence have indicated that CASP6 mediates the activation of innate immunity and inflammasomes, and can promote the activation of programmed cell death, including pyroptosis, apoptosis, and necroptosis [35]. GSDMA can act as a regulator of programmed cell death [36, 37]. Studies have reported that GSDMA may be a tumor suppressor gene [38–40], which is generally suppressed in esophageal squamous cell carcinoma and gastric cancer. CHMP4C (chromatin-modifying protein 4 C) plays a role in cell division, which prevents the accumulation of DNA damage by delaying abscission [41–43]. The polymorphism of CHMP4C increases the susceptibility to cancer and might promote genome instability, thereby inducing cancer [20]. Li et al. found that CHMP4C can increase the NSCLC cells’ survival ability after ionizing radiation, and its silencing can increase the sensitivity of the cells to radiation [44]. Compared to the normal tissues, CHMP4C is up-regulated in cervical cancer and lung squamous cell carcinoma, the knockdown of CHMP4C inhibits the proliferation of the cancer cells [21, 45]. Similar to the results of our study, the high expression of CHMP4C might be related to the poor prognosis of osteosarcoma. Through the analysis of multiple public databases, CHMP4C was found to be up-regulated in a variety of tumors, including osteosarcoma. Consistent with this, RT-qPCR was performed to validate the high expressed CHMP4C in the osteosarcoma cell lines. We found that overexpression of CHMP4C enhanced the migratory and invasive abilities of osteosarcoma cells. These results indicate that PRGs play an important role in tumors, promoting or inhibiting metastasis and progression. Moreover, CHMP4C might act as a cancer-promoting factor, which is expected to become an effective target for cancers.

We also established a PRLs prognostic signature for osteosarcoma patients. Firstly, to determine the PRLs, we performed Pearson correlation analysis between the PRGs and lncRNA. By differential expression analysis, we get the differentially expressed PRLs. Next, the differentially expressed PRLs related to the prognosis were selected, and a 9 PRLs signature was developed using the LASSO Cox analysis. As shown by the risk model, the prognosis of the high-risk patients was found to be significantly lower than that of the low-risk patients. The GSEA results suggest that the immune-related functions are enriched in the low-risk patients, suggesting that immune regulation might be related to the improvement of prognosis.

LncRNAs usually do not encode proteins, but they are important in gene regulation and cell metabolism [46]. Recent studies have shown that lncRNAs are involved in the pathological progression of cardiovascular diseases, tumors, neurological diseases, and other diseases by directly or indirectly acting on the pyroptosis-related pathways [47–50]. Nevertheless, the research on lncRNA related to pyroptosis in cancer, especially osteosarcoma, is very inadequate. We have identified 9 PRLs for constructing the risk model, some of which have been reported to be related to tumors. FOXD2-AS1 is up-regulated in a variety of cancers and has been identified as an oncogene [51–53]. The knockdown of FOXD2-AS1 in osteosarcoma has been found to inhibit tumor growth and invasion in vitro and vivo [54, 55], and inhibit its resistance to cisplatin [56]. AL035446.1 might serve as a pro-cancer factor for clear cell renal cell carcinoma patients in the lncRNA risk signature constructed by Yang et al[57]. The UNC5B-AS1 functions similarly to FOXD2-AS1, and its expression is up-regulated in hepatocellular carcinoma, papillary thyroid cancer, and prostate cancer [58–60]. The silencing of UNC5B-AS1 inhibits tumor growth [61, 62], but it has not been reported in osteosarcoma. SENCR has been extensively studied in the vascular smooth muscle cells and endothelial cells [63, 64], but recent studies have showed that it also has a role in cancer. Cheng et al. reported that SENCR promotes the cell proliferation and progression of the NSCLC cells through sponge miR-1-3p [65]. According to the prognostic model constructed by Guo et al., AC090559.1 is considered to be related to ferroptosis and is a favorable prognostic factor in lung adenocarcinoma [66]. The functions of AC010894.2, AC018904.1, BX322562.1, AC016596.1 have not been reported in the literature. Our study proved their relationship with the prognosis of patients with osteosarcoma and inferred their role in osteosarcoma through enrichment analysis. The role of these lncRNAs in osteosarcoma needs to be further explored in the experimental studies.

This study still has certain limitations. Firstly, there are currently few public gene expression databases containing prognostic information for the patients with osteosarcoma, resulting in a small number of tumor samples in our study. In the future, a more accurate prognostic model should be built using a larger sample size. Secondly, the clinical information of the data set is not complete, and more abundant clinical data are needed to evaluate the relationship between the model and the clinic. Finally, the exact mechanism underlying how CHMP4C promotes proliferation, invasion and migration also requires further exploration. Hence, further functional experimental research is warranted in the future.

In summary, this study constructed a pyroptosis-related markers signature in osteosarcoma, which is of great significance in determining the prognosis of osteosarcoma patients. The results of this study have emphasized the importance of pyroptosis-related markers to osteosarcoma and provided important evidence for revealing the pathogenesis of osteosarcoma and guiding the future treatment of osteosarcoma.

## Supplementary Information

**Additional file 1: Table S1**. Risk coefficients of three PRGs. **Table S2**. Risk coefficients of six PRLs. **Table S4**. Primers used in this study.

**Additional file 2: Table S3**. The 57 PRGs

**Additional file 3: Fig. S1**. A, B PCA based on the six pyroptosis-related genes signature. C, D The relationship between the risk score and metastasis. E-G The nomogram calibration curves for predicting 1-, 3-, and 5-year survival in the GSE21257 cohort. H Kaplan–Meier analysis based on the pan-cancer data set and univariate Cox regression analysis of CHMP4C. I The expressions of CHMP4C in tumor and adjacent normal tissues.

**Additional file 4: Fig. S2**. The PPI network.

## Data Availability

The datasets supporting the conclusions of this article are available in the TARGET (https://portal.gdc.cancer.gov/), Gene Expression Omnibus (GEO) database (https://www.ncbi.nlm.nih.gov/geo/), Genotype-Tissue Expression (GTEx) database (https://gtexportal.org/).
