# Supplementary material for: Identification of pyroptosis-related genes and long non-coding RNAs signatures in osteosarcoma
Source: Cancer Cell Int. 2022 Oct 16;22:322. doi: 10.1186/s12935-022-02729-1 (PMC9575257; doi:10.1186/s12935-022-02729-1)
Supplement: Supplementary file 1 — Additional file 1: Table S1. Risk coefficients of three PRGs. Table S2. Risk coefficients of six PRLs. Table S4. Primers used in this study. [file 12935_2022_2729_MOESM1_ESM.docx]

| Gene | Coefient |
| --- | --- |
| CHMP4C | 0.0989364172871374 |
| GZMA | -0.00405571594070448 |
| BAK1 | -0.0550377391331656 |
| GSDMA | -0.602811665856014 |
| CASP1 | -0.0995357348588547 |
| CASP6 | -0.167804307140446 |

**Table S1 Risk coefficients of six PRGs.**

PRGs: pyroptosis-related genes.

| Gene | Coefient |
| --- | --- |
| AC090559.1 | -0.709483836074113 |
| AC010894.2 | -0.475062678794469 |
| FOXD2.AS1 | -0.218033357015728 |
| BX322562.1 | -0.124789627493896 |
| AL035446.1 | -0.111570036729204 |
| AC016596.1 | 0.049957357604976 |
| AC018904.1 | 0.098333143397259 |
| UNC5B.AS1 | 0.467180342104728 |
| SENCR | 0.869352393113547 |

**Table S2 Risk coefficients of nine PRLs.**

PRLs: pyroptosis-related lncRNAs.

| Gene | Direction | Sequences (5’ to 3’) |
| --- | --- | --- |
| CHMP4C | Forward | AGAAGCCCTGGAGAACTCAC |
|  | Reverse | CTTGGGCAGTATCCTGTTGC |
| GAPDH | Forward | GAAGGTCGGAGTCAACGG ATTTG |
|  | Reverse | ATGGCATGGACTGTGGTCATGAG |

**Table S4 Primers used in this study.**
