# Supplementary figures and images for: Identification of pyroptosis-related genes and long non-coding RNAs signatures in osteosarcoma
Source: Cancer Cell Int. 2022 Oct 16;22:322. doi: 10.1186/s12935-022-02729-1 (PMC9575257; doi:10.1186/s12935-022-02729-1)

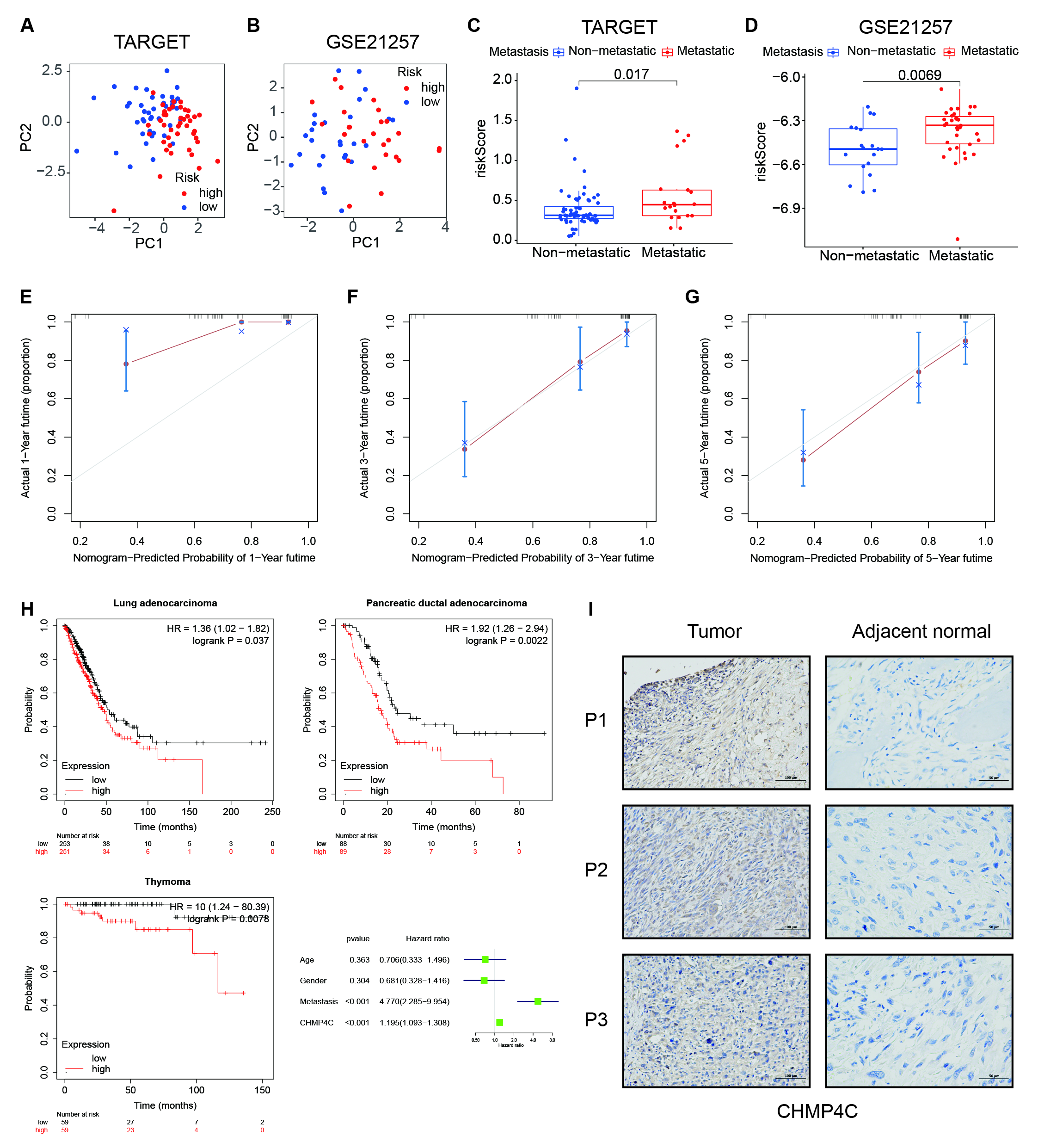

Supplement: Supplementary file 3 — Additional file 3: Fig. S1. A, B PCA based on the six pyroptosis-related genes signature. C, D The relationship between the risk score and metastasis. E-G The nomogram calibration curves for predicting 1-, 3-, and 5-year survival in the GSE21257 cohort. H Kaplan–Meier analysis based on the pan-cancer data set and univariate Cox regression analysis of CHMP4C. I The expressions of CHMP4C in tumor and adjacent normal tissues. [file 12935_2022_2729_MOESM3_ESM.tif]

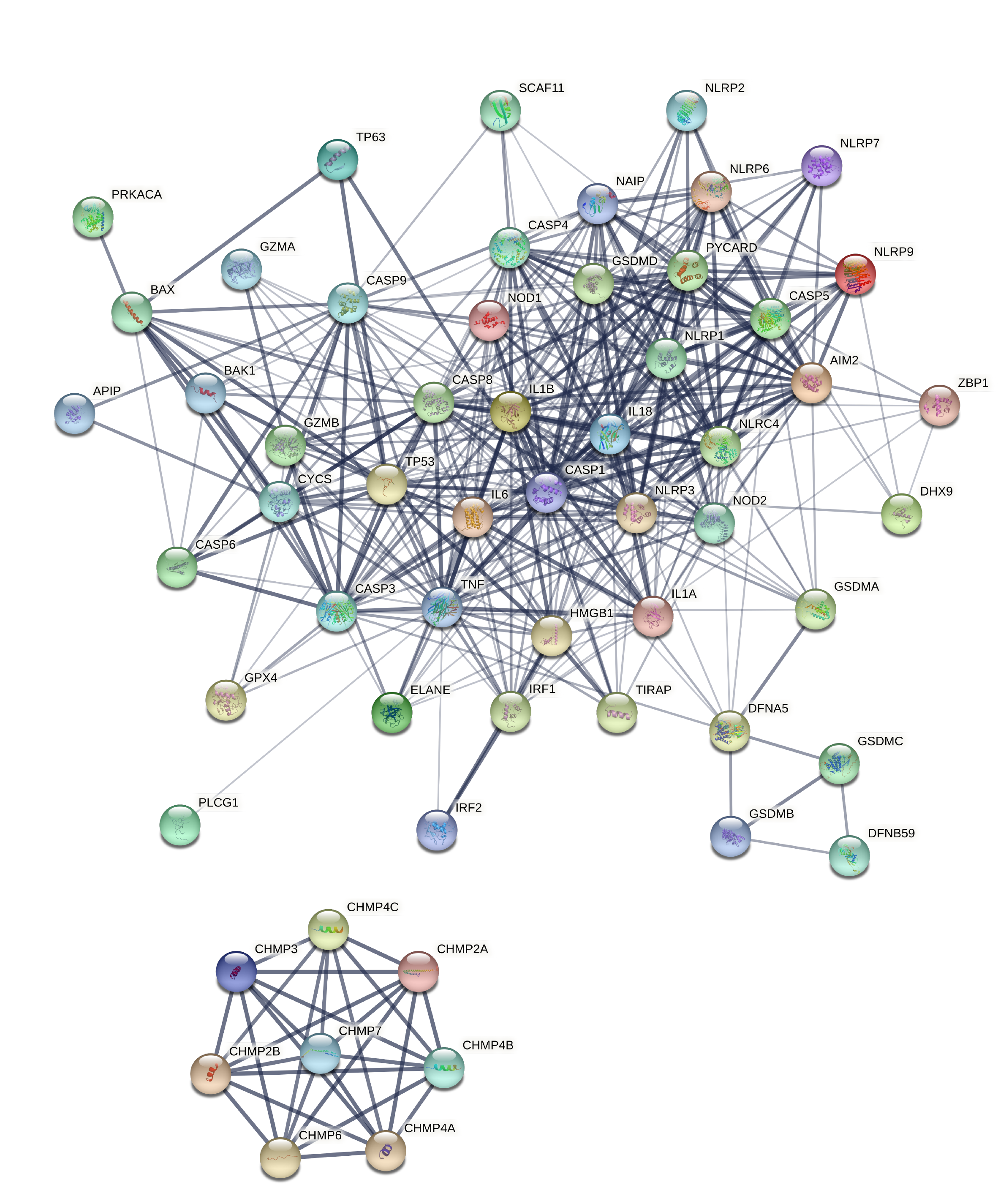

Supplement: Supplementary file 4 — Additional file 4: Fig. S2. The PPI network. [file 12935_2022_2729_MOESM4_ESM.tif]
